# Notes on Preparations for the Sick

**Published:** 1897-06

**Authors:** 


### Notes on Preparations for the Sick.

**Colchicine Salicylate Capsules** (TROCHET).—B. KUHN, London.—Each of these capsules contains quarter of a milligramme of colchicine dissolved in 20 centigrammes of salicylate of methyl, from betula lenta. They are made by Trochet. of Paris, and are as excellent in appearance as the combination of colchicum and methyl salicylate should be in action. Eight to fifteen capsules may safely be given in the day, and should give more trustworthy and uniform results than the ordinary preparations of colchicum and artificial salicylic acid.

**Apenta.**—THE APOLLINARIS CO., LIMD., London.—The Apollinaris Company have now acquired the control of another natural bitter water drawn from springs at Buda Pest. The following analysis has been supplied to us:—

Parts per 1000.

| | | | |
|---|---|---|---|
| Sulphate of Magnesia ... | 24.4968 | Bicarbonate of Lime ... | 0.8843 |
| Sulphate of Soda ... ... | 15.4320 | Bicarbonate of Protoxide of Iron ... ... ... ... | 0.0189 |
| Sulphate of Lime ... ... | 1.0989 | Silicic Acid ... ... ... | 0.0100 |
| Chloride of Sodium ... | 1.8720 | | |

The springs have been placed under the absolute control of the Royal Hungarian Chemical Institute. It will be seen that the sulphates of magnesia and soda are the principal ingredients. The water is agreeable to the palate, and is a most efficient saline aperient.

---

**Humanized Milk; Peptonized Milk; Sterilized Milk; Muffler's Food; Whey.**—THE AYLESBURY DAIRY COMPANY LIMITED, London.—This well-known firm has for a considerable time prepared a milk to take the place of the baby's natural food. The samples of **Humanized Milk**, which came to us in air-tight bottles, we found perfectly sweet, even after keeping some time. This is an important point if a supply is needed at some distance from London. Its composition has been proved by analysis to as nearly resemble maternal milk as an artificial production can; and we can recommend it as a nourishing and sound food for infants.

The **Peptonized Milk** is also perfectly sterilised; but we detected the slight peculiar taste so often noticed in these predigested milks. The infants to whom we gave it took it readily, and throve well on it.

The **Sterilized Milk**, prepared without the addition of any chemical body, is an exceedingly valuable preparation. The demand for this preparation should be great; for we believe that the profession is awakening to the value of sterilised milk. The milk has practically no "scalded" taste.

If the analysis of **Muffler's Food** given by the Company is correct—and we do not for a moment doubt it, though we have not tested it in the laboratory—it is, perhaps, the best of these foods in the market. The carbohydrates are present in very large proportion; and the percentage of albuminoids and fats is remarkably high. This food must certainly be more nourishing than many that are more commonly used.

The demand for **Whey** is not, we believe, great; but as this variety is prepared without the addition of acid, and is free from fat and casein, it should supply a want.

---

**Beef Juice.**—ARMOUR & Co., London.—This beef juice has a dark colour, a viscid consistence, and when diluted five times has a gravity over 1030, with soluble albumen amounting to 0.5%, as estimated by the picric acid tube, so that the undiluted fluid contains 2.5% of soluble albumen. There appears to be no excess of salt. Diluted with soda water, sherry, or port, it forms a most nourishing and acceptable drink for those invalids who cannot take more solid foods, and whose debility necessitates the use of concentrated soluble food.

---

**Peptarnis.**—LIEBIG'S EXTRACT OF MEAT COMPANY, LIMITED, London.—This preparation contains nearly 50% of albuminous matter, consisting practically of equal amounts of albumose and peptone, the total nitrogen amounting to nearly 10%. It is quite free from the unpleasant taste and odour which have commonly been associated with the presence of peptones, and it does not contain any deleterious preservatives. We have in this preparation a concentrated, digested, soluble food, with a pleasant aroma and a flavour which is not likely to nauseate even the most fastidious invalid. It is a brilliant success, and should soon become popular.

As an indication that the Liebig's Extract of Meat Company still maintain a leading position as manufacturers of invalid foods, we are glad to observe that at a meeting of the Harben Nomination Committee, held recently in connection with the British Institute of Public Health, Professor Max von Pettenkofer, Scientific Director of Liebig's Extract of Meat Company, was nominated Harben Gold Medallist for 1897. The medal was founded in 1895 by Henry Harben for the recognition of eminent service to the public health, and one presentation is made every year.

---

**Litmus Pencil.**—THOS. CHRISTY & Co., London.—This pencil is constructed of pure blue and red litmus inserted at the two ends of an ordinary wood pencil. It is said to be capable of detecting one part in a hundred thousand, whereas the ordinary litmus paper can only detect one part in fourteen hundred. For use, the ends are sharpened—the one for acids, the other for alkalies; the pencil is moistened, and with it are made marks on a strip of white paper, which is inserted into the fluid to be tested. We have found that it acts quite in accordance with expectations.

